# Role played by Th2 type cytokines in IgE mediated allergy and asthma

**DOI:** 10.4103/0970-2113.63609

**Published:** 2010

**Authors:** Sudha S. Deo, Kejal J. Mistry, Amol M. Kakade, Pramod V. Niphadkar

**Affiliations:** *Medical Research Society, Raja Ram Mohan Roy Road, Prathna Samaj, Girgaum, Mumbai - 400 004, India*; 1 *Sir H. N. Hospital and Research Centre, Raja Ram Mohan Roy Road, Prathna Samaj, Girgaum, Mumbai - 400 004, India*

**Keywords:** Allergy, asthma, cytokines, Type 2 helper T cells

## Abstract

**Objective:**

Recent evidence suggest that allergen type 2 helper T cells (Th2) play a triggering role in the activation/recruitment of IgE antibody producing B cells, mast cells and eosinophils. Reduced microbial exposure in early life is responsible for a shift of Th1/Th2 balance in the immune system towards the pre-allergic Th2 response. The Th1 predominantly produce IFNγ and delayed type hypersensitivity while Th2 secrete IL-4, IL-5, IL-6, IL-13 and regulate B cell and eosinophil mediated responses. To assess regulatory changes in the immune system, in patients with allergy and asthma, we studied the cytokine profile in serum in comparison with normal healthy controls.

**Patients and Methods:**

A total of 170 patients with various allergies and asthmatic conditions were studied, for cytokines in the serum by ELISA using kits from Immunotech, and analyzed to identify the triggering factors or main contributors towards allergy and asthma.

**Results:**

Our study showed increase in the levels of IL-4, IL-5 and IL-6 in all groups which were non- significant. But the levels of IL-10, IL-13 and TNF α were highly significant. Besides, we found correlation of GM-CSF with IL-10. Significant correlation with different cytokines was observed. Most of these patients showed increase in IgE levels.

**Conclusions:**

This study gives a better understanding of how cytokines are the mediators of balance of Th1 and Th2 immune responses and IgE synthesis is controlled by cytokines. Further studies will eventually lead to improved treatment strategies in the clinical management of IgE mediated allergy.

## INTRODUCTION

It has been proposed that reduced microbial exposure in early life leads to polarization of allergen-specific T-cell memory towards the Th2 instead of the Th1 immune response. Whether reduced microbial exposures are the only environmental stimuli influencing this immune effect is unclear, Th1 and Th2 subsets develop from the same precursor cells, via activated antigen-presenting cells under the influence of naıve CD4^+^T lymphocytes, and the pattern of differentiation is determined by environmental stimuli present early during immune responses.[1] Activated Th2 lymphocytes produce IL-4, IL-13, and IL-5, which are responsible for IgE production by B cells, eosinophil activation and recruitment, and mucus production. In contrast, Th1 cells differentiate from naıve CD4^+^ cells in response to microbial activation of antigen-presenting cells under the influence of IL-12. Differentiated Th1 cells secrete interferon-γ, which is important in intracellular destruction of phagocytosed microbes. Furthermore, interferon-gamma produced by Th1 cells and IL-4 produced by Th2 counter-regulate each other.[2]

Failure of an immune deviation from an allergen specific Th2 response to a Th1 immune response has been proposed as the mechanism responsible for the increased allergic disease prevalence associated with reduced microbial exposure in early life. Recent evidence suggests that dysregulation in the immune system involved in allergy and asthma cannot be explained simply by the Th1/Th2 dichotomy. Another mechanism may involve T-regulatory/suppressor cells. It has been shown that maternal T-regulatory cells act to suppress auto-immune responses and create an immune homeostasis in the feto-maternal relationship.[3]

The initial view is that reduced microbial stimulation of cells of innate immunity, as a result of improved hygiene, causes a reduction of Th1 polarization and therefore reduced interferon-γ. As a result, a Th2-dominant immune response is observed.[4] However, recent evidence suggests that reduced microbial exposure also leads to reduced stimulation.[5] It is most likely that the combination of reduced Th1 cytokines (interferon-γ, and IL-12) and reduced T-regulatory cytokines (IL-10 and TGF-β) secondary to reduced microbial burden, in early life, is responsible for the Th2-skewed immune response. Understanding early-life immune mechanisms responsible for atopic diseases continues to be an exciting area of research, but further work is needed to determine how cytokines of T-regulatory cells act to balance the Th1 and Th2 immune response.[6]

The cytokines which are involved in the mechanisms in asthma need to be revealed. Hence we undertook the study of these cytokines to identify the nature of the immune responses in our population-either Th1 type or the Th2 immune response that brings about the changes due to environmental exposure. Cytokines are categorized by their major specific function(s). IL-4 causes a switch to IgE production by differentiating B cells. IFNγ can inhibit that switch and prevent the production of specific IgE. IL-10 can actually inhibit the activity of IFNγ, allowing the original IL-4 to proceed in the IgE cascade. Thus, an allergic response can be viewed as an allergen-specific production of excess IL-4 and/or IL-10, lack of adequate IFNγ production or both. Eosinophilic inflammation, a major component of allergic reactions, is under control of IL-5 and TNF α, Depending on the intensity of exposure to allergen it can influence the cytokine profile. The understanding of the allergen-specific TH1/TH2 functional rating and its importance in the patho-physiology of allergic diseases may also have utility in clinical monitoring situations. Research related to allergic inflammation and cytokines continues to move steadily from bench to bedside. The primary inflammatory lesion of asthma consists of accumulation of CD4^+^ T helper type 2 (TH2) lymphocytes and eosinophils in the airway mucosa. TH2 cells orchestrate the asthmatic inflammation through the secretion of a series of cytokines, particularly IL-4, IL-13, IL-5, and IL-9. IL-4 is the major factor regulating IgE production by B cells, and is required for optimal TH2 differentiation. Cytokines are of particular importance for mast cell recruitment, development, and function.[7]

## PATIENTS AND METHODS

### Patients

Peripheral blood was collected in plain tubes from 170 selected patients. These patients were selected on the basis of their allergic and asthmatic conditions. The detailed history was taken and proforma was filled. Informed consent was taken from each patient. These patients were off treatment during the collection of blood. Most of the patients were treated with anti-histamines and they were informed prior to blood collection.

Ethical approval was taken from the hospital management for collection of the samples and conduction of the project.

### Controls

A total of 24 patients were selected from a group of healthy laboratory individuals without any ailments during the past few months.

### Methodology

Blood was collected in plain tubes without any anti-coagulant and allowed to settle for 15 minutes for clotting and serum to be separated. Serum, thus separated, is then collected by centrifugation and stored in allocates in eppendorf tubes and preserved in minus 70ºC until assay. ELISA is performed for the detection of cytokines such Il-4, IL-5, IL-6, IL-10, IL-13, TNF α and GM-CSF. ELISA kits from Immunotech (France) were used which involved sandwich type assay. The intensity of coloration was proportional to the concentration of the cytokine in the serum.

Briefly, 100ul of the calibrator or the sample is added to the wells of a 96 well ELISA plate coated with the respective monoclonal antibody to the cytokine of interest, incubated for 2 hr at 18-25ºC with shaking. After 3 × washes, 50 ul of biotnylated antibody and 100ul of streptavidin-HRP conjugate at appropriate dilution and incubated for 30 minutes at 18-25ºC with constant shaking. After 3 × washes, 100 ul of substrate is added to the test wells and further incubated for 20 minutes at 18-25ºC with constant shaking. After the incubation 50 ul of stop solution was added to end the reaction. The color reaction is then read at 450 nm using ELISA reader. Depending on the assay the biotnylated conjugate is diluted according to the kit insert. The assay was performed according to the protocol given in the kit insert.[8,9]

The sample results were calculated by interpolation from a calibrator curve performed in the same assay as that of the sample. The curve was drawn by plotting the cytokine concentration of the calibrator on the horizontal axis and the absorbance on the vertical axis. From this plot the absorbance of the sample was then read by using the corresponding absorbance of the test well. Inter and intra assay precision was thus determined.

### Statistical analysis

The results were analyzed on SPSS version 15 and student '*t*' test was used for evaluation of statistical significance. Spearman's coefficient correlation has been used for comparison with different parameters and one way ANOVA test for significant correlation between groups.

## RESULTS

From our studies we observed that most of our patients were in the age group 25-45 years. Age differences in different groups were not seen. There was also no significant difference as far as the duration of the disease is concerned. Comparison of total WBC count did not show any significant changes in percentage. Significant or increased eosinophil percentage was seen when compared to controls. Significant increase in Total IgE was observed in different allergic groups when compared to normals (*P* less than 0.001)[Table 1].

**Table 1:** Data showing general characteristics of patients with total IgE

Group	Age (yrs)	Sex M/F	Duration months	Total Total WBC × 10^9^/l	Total eosinophil %	Total IgE IU/ml
Normals 24	34.04 ± 2.02	13/11	-	8.98 ± 0.42	4.17 ± 0.72	176.50 ± 22.63
Asthma 20	2950 ± 3.23	9/11	98.40 ± 15.85	10.75 ± 0.77	6.00 ± 0.96	2115.98 ± 766.67
Allergic rhinitis 57	35.03 ± 2.02	24/33	70.35 ± 10.07	9.69 ± 0.41	5.41 ± 0.65	1126.58 ± 211.217
Urticaria 58	36.30 ± 2.042	27/31	62.15 ± 8.07	9.03 ± 0.46	4.58 ± 0.52	910.97 ± 229.63
Dermititis 10	44.30 ± 3.49	3/7	52.60 ±18.60	8.77 ± 1.51	6.4 ± 1.50	2818.75 ± 1867.74
Both 25	34.63 ± 3.1	12/13	72.74 ± 12.61	9.35 ± 0.63	5.05 ± 0.91	1263.08 ± 312.06

Values are expressed as Mean ± SE

To assess the regulatory changes in the immune system in patients with allergy and asthma we studied the cytokine levels in the serum and compared the levels with the normal controls as well as in different allergic groups. Cytokines derived from Th1 and Th2 type cells that included IL-4, IL-5, IL-6, IL-10, IL-13, TNF α and GM-CSF studied using ELISA kits from Immunotech (France). Levels of cytokines in the allergic were compared with that of healthy controls as shown in Table 2.

**Table 2:** Quantitation of cytokines by ELISA in different allergic groups

	Normals (24)	Asthma (20)	Allergic rhinitis (57)	Urticaria (58)	Dermitis (10)	Allergic rhinitis and dermititis (25)
IgE	176.50 ± (14-390)	2116 ± 767**	1142 ± 214.31	910.97 ± 229.63	2818.8 ± 1867.7	1263.08 ± 312.06
		(27-15600)	(8-8400)**	(8-11550)**	(225-19200**	(8-5800)**
IL-4	3.04 ± 0.53 (0-8)	4.8 ± 1.33	5.33 ± 0.59	5.93 ± 0.653	5.10 ± 1494	4.88 ± 0.809
		(0-22)	(0-22)*	(0-18)*	(0-13)	(0-14)
IL-5	3.63 ± 0.59 (0-8)	4.35 ± 0.79	4.39 ± 0.39	3.66 ± 0.32	3.15 ± 0.58	3.88 ± 0.57
		(0-15)	(0-15)	(0-13)	(0-5)	(0-13)
IL-6	19.38 ± 6.85 (0-150)	17.10 ± 2095	19.85 ± 5.34	22.87 ± 6.24	15.85 ± 3070	21.80 ± 11.29
		(0-51)	(0-285)	(0-285)	(0-36)	(0-285)
IL-10	43.54 ± 9.22 (0-150)	54.20 ± 12.52	89.07 ± 14.11	77.36 ± 12.67	58.80 ± 12.17	89.76 ± 26.01
		(0-246)	(0-630)	(0-630)	(12-124)	(0-630)
IL-13	10.37 + 1.15 (0-27)	34.53 ± 14.19	14.11 ± 14.99	8.87 ± 2.048	5.60 ± 0.909	5.40 ± 0.76
		(0-214)	(0-214)**	(0-110)**	(2-10)**	(0-18)**
TNF α	40.21 + 6025 (0-131)	118.38 ± 48.29	70.74 ± 17.59	60.34 ± 16.14	30.8 ± 5.020	46.30 ± 4.94
		(12-910)	(9-910)	(0-950)	(8-58)	(9-120)
GM-CSF	6.88 + 1.42 (1.5-34)	17.40 ± 7.81	26.34 ± 10.32	2843 ± 10.63	38.00 ± 34.11	41.13 ± 22.53
		(1.5-134)	(1.5-442)	(1.5-442)	(1.5-345)	(1.5-442)

Significant values

* *P* < 0.01;

** *P* < 0.001 cytokines values are in pg/ml, IgE IU/ml

From Table 2 it is observed that in the group of patients showing Allergic Rhinitis IL-4 and IL-13 cytokines are significantly increased as compared to normals, while only IL-13 is significantly increased in patients showing Urticaria, Dermitis and those showing symptoms with respiratory as well as skin problems.

From our studies we can see that functional analysis of the role of cytokines seems to be main factor in determining the degree of the airway inflammation and hyperresponsiveness. It would thus appear that cytokine production rather than influx of eosinophils or the production of IgE is the cause of allergic conditions.

From Table 3, analysis of our normal data shows that there should be an association of IL-4 cytokine with that of IL-5 and a negative correlation of of IL-5 with that of IL-10 and a positive correlation of TNF α with IL-6 and IL-13.

**Table 3:** Association of individual cytokines in the presence of other cytokines; analysis by Spearman's correlation

	Total IgE	IL-4	IL-5	IL-6	IL-10	IL-13	TNF α	GM-CSF
A-Normals N= 24								
Total IgE	1	–0.092	–0.050		0.006	–0.380	–0.091	–0.152
IL-4	–0.092	1	0.696**	–0.086	–0.382	0.102	0.052	0.165
IL-5	–0.050	0.696**	1	0.035	–0.646**	–0.028	–0.104	0.272
IL-6	0.195	–0.086	0.035	1	–0.040	0.142	0.478*	–0.182
IL10	0.006	–0.382	–0.646**	–0.040	1	0.074	0.062	–0.206
IL13	–0.380	0.102	–0.028	0.142	0.074	1	0.513*	–0.009
TNF α	–0.091	0.052	–0.104	0.478*	0.062	0.513*	1	–0.268
GM-CSF	–0.152	0.165	0.272	–0.182	–0.206	–0.009	–0.268	1
B-Allergic rhinitis								
Total IgE	1	0.120	0.132	0.034	0.082	0.043	–0.018	–0.084
IL-4	0.120	1	0.436*	0.012	0.077	0.526**	0.527**	0.023
IL-5	0.132	0.436**	1	0.155	0.508**	0.332*	0.257	–0.032
IL-6	0.034	0.012	0.155	1	–0.091	0.035	0.133	0.044
IL10	0.082	0.077	0.508*	–0.091	1	0.134	0.068	0.071
IL13	0.043	0.526**	0.332*	0.035	0.134	1	0.911**	0.212
TNF α	–0.018	0.527**	0.257	0.138	0.068	0.911**	1	0.224
GM-CSF	–0.084	0.023	–0.032	0.044	0.071	0.212	0.224	1
C-Asthma								
Total IgE	1	0.120	–0.200	–0.216	–0.281	0.210	–0.105	–0.037
IL-4	0.120	1	0.521*	0.475*	0.208	0.760*	0.751*	0.773*
IL-5	–0.200	0.521*	1	0.335	0.529*	0.419	0.387	0.467*
IL-6	–0.216	0.475*	0.335	1	0.108	0.178	0.360	0.304
IL10	–0.281	0.208	0.529*	0.108	1	0.530*	0.496*	0.567**
IL13	0.210	0.760**	0.419	0.178	0.530	1	0.864**	0.941**
TNF α	–0.105	0.751**	0.387	0.360	0.496*	0.864**	1	0.959
GM-CSF	–0.037	0.773**	0.467*	0.304	0.567**	0.941**	0.959**	1
D-Dermitis								
Total IgE	1	0.674*	0.226	0.163	–0.355	0.405	0.437	–0.124
IL-4	0.674*	1	0.642*	0.319	–0.357	0.322	0.701*	0.289
IL-5	0.226	0.642*	1	0.091	–0.116	–0.083	0.172	–0.029
IL-6	0.163	0.319	0.091	1	–0.280	0.200	0.005	0.430
IL10	–0.355	–0.357	–0.116	–0.280	1	0.287	–0.033	–0.435
IL-13	0.405	0.322	–0.083	0.200	0.287	1	0.383	–0.013
TNF α	0.437	0.701*	0.172	0.005	–0.033	0.383	1	0.415
GM-CSF	–0.124	0.289	–0.029	0.430	–0.435	–0.013	0.415	1
E-Urticaria								
Total IgE	1	–0.140	–0.121	–0.059	0.097	–0.086	–0.080	–0.069
IL-4	–0.140	1	0.504*	–0.013	–0.132	0.256	–0.082	–0.110
IL-5	–0.121	0.504**	1	0.073	0.535**	0.003	–0.072	–0.071
IL-6	–0.059	–0.013	0.073	1	–0.037	0.250	0.649**	0.029
IL10	0.097	–0.132	0.535**	–0.037	1	–0.020	0.093	0.038
IL13	–0.086	0.256	0.003	0.250	–0.020	1	0.377**	–0.001
TNF α	–0.080	–0.082	–0.072	0.649**	0.093	0.377**	1	–0.082
GM-CSF	–0.069	–0.110	–0.071	0.029	0.038	–0.001	–0.082	1
F-Skin and rhinitis								
Total IgE	1	0.097	–0.102*	0.092	–0.218	–0.071	0.028	–0.158
IL-4	0.097	1	0.333	0.148	–0.095	0.128	0.099	–0.203
IL-5	–0.102	0.333	1	0.226	0.760**	–0.039	0.023	–0.153
IL-6	–0.092	0.148	0.226	1	–0.132	0.129	0.645**	0.023
IL10	–0.218	–0.095	0.760**	–0.132	1	0.106	–0.157	0.050
IL13	–0.071	0.128	–0.039	0.129	0.106	1	–0.036	–0.117
TNF α	0.028	0.099	0.023	0.645**	–0.157	–0.036	1	–0.097
GM-CSF	–0.158	–0.203	–0.159	0.023	0.050	–0.117	–0.097	1

Significant values

* *P* < 0.01

** *P* < 0.001, cytokines values are in pg/ml, IgE IU/ml

Studies conducted on allergic rhinitis showed that there is an association of IL-4 with significant increase in IL-13 and TNF α. Similarly IL-5 is significantly associated with IL-4, IL-10 and IL-13.

In asthma we found a positive association of IL-4 with IL-5, and significant association with IL-13, TNF α as well as GM-CSF, while IL-5 is positively associated with IL-4, IL-10 and GM-CSF. There is strong association of IL-13 with TNF α and GM-CSF.

In dermititis there is positive correlation of IL-4 with IgE and TNF α only. In patients showing urticaria, there is a positive correlation of IL-4 with IL-5, while that of IL-5 with IL-10. Similarly there is association of TNF α with IL-6 and IL-13. In patients with both types of allergy i.e. skin and rhinitis, there was a positive correlation of IL-5 with IL-10 and that of IL-6 with TNF α.

Table 4 depicts the significant levels of cytokines compared between different groups as analysed by ANOVA test. From this Table we observed significant levels of IL-13 between groups. Similarly total IgE is also found to be significantly correlated within the groups with *P* less than 0.01.Thus our studies show significant correlation of IL-13 as the main cytokine orchestrating the total IgE production.

**Table 4:** Significant levels of cytokines compared between groups as analyzed by ANOVA test

	Sum of squares	df	Mean square	F	Sig.
IL-4					
Between groups	147.849	5	29.570	1.406	0.224
Within groups	3954.089	188	21.032		
Total	4101.938	193			
IL-5					
Between groups	28.564	5	5.713	0.725	0.605
Within groups	1480.812	188	7.877		
Total	1509.376	193			
IL-13					
Between groups	12615.313	5	2523.063	2.764	0.020*
Within groups	170674.405	187	912.697		
Total	183289.718	192			
GM-CSF					
Between groups	17514.378	5	3502.876	0.605	0.696
Within groups	1048693.100	181	5793.885		
Total	1066207.479	186			
TNF					
Between groups	94899.439	5	18979.888	1.286	0.271
Within groups	2773862.742	188	14754.589		
Total	2868762.182	193			
IL-6					
Between groups	869.665	5	173.933	0.100	0.992
Within groups	326793.713	188	1738.264		
Total	327663.378	193			
IL-10					
Between groups	52079.344	5	10415.869	1.158	0.332
Within groups	1691628.434	188	8998.024		
Total	1743707.778	193			
Total IgE					
Between groups	43746219.128	5	8749243.826	2.655	0.024*
Within groups	619559188.528	188	3295527.599		
Total	663305407.656	193			

* *P* < 0.01

**Figure 1:**
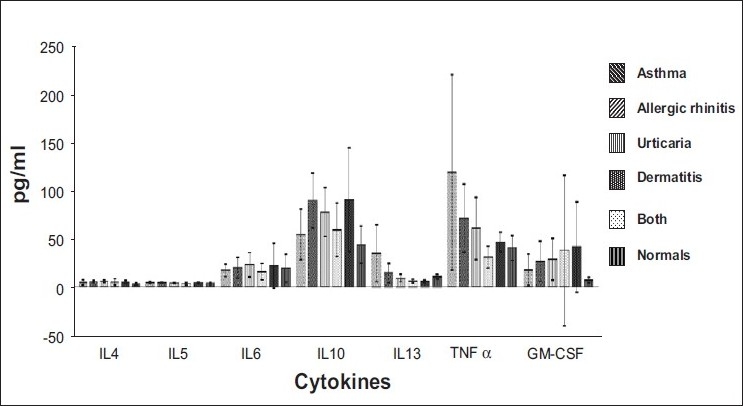
Levels of cytokines in different allergic conditions

Figure 1 gives a comparative study of all the cytokines in different disease conditions. The levels of IL-10 and TNF α increased significantly compared to other cytokines in all the allergic conditions.

Our studies show the importance of IL-4 and IL-5 cytokines in response to allergy. We observed an increase in the IL-4 as well as IL-5 cytokine production which clearly shows the association of both the cytokines in allergic conditions and their important role with respect to hypersensitivity reactions. Since the individuals are from Mumbai-based population they are exposed to environmental pollution which is very high in this urban area and hence there is increase in allergic immune response with consequently increase in IgE immunoglobulin. This increase is higher than the normal level observed in the western population. Hence our study could reveal the role of Th2 cytokines in hypersensitivity reactions.

## DISCUSSION

We found that there is a strong association of IL-4 cytokines with IL-5 in most of cases of allergic rhinitis, asthma, urticaria as well in patients showing skin and rhinitis. This shows that IL-4 is the main cytokine that brings about the increase in IgE synthesis. IL-10 is also associated with IL-5 in urticaria as well as those showing skin and rhinitis. The association of IL-13 with TNF α was seen only in urticaria patients. While GM-CSF shows its presence in urticaria as well as allergic rhinitis along with IL-13. These studies confirm the importance of IL-5 in eosinophilic inflammation in man, but question's the role of eosinophils in asthma. IL-13 has many actions similar to those of IL-4 and also regulates IgE production but, unlike IL-4, it does not regulate T cell differentiation to Th2 cells and T lymphocytes do not respond to IL-13.

Cytokines are important in the chronic inflammation of asthma and play a critical role in orchestrating the allergic inflammatory response. Of particular importance to allergic disease is the recent recognition of the regulation of helper immune function by two lineages of T helper cells, i.e., Th1 and Th2, by these cytokines.[10] The Th2 hypothesis of allergy considers atopy as a Th2-driven hypersensitivity reaction to allergens of complex genetic and environmental origins, in which the Thl lineage, normally driven by IL-2, TNF, and IFN-γ is deficient, and in which a predominant Th2 response is seen that is driven by IL-4, IL-13, IL-5, and IL-10. This knowledge is finding application in both the diagnosis and therapy of allergic diseases, through the measurement or use of cytokines, which may replace deficient quantities, or the use of anti-cytokines, which may neutralize elevated quantities of cytokines, events that collectively contribute to the immunologic imbalance characteristic of the allergic state.[11] In future, the application of cytokines will continue to find clinical application in allergic disease, and it behaves the clinical allergist-immunologist to keep abreast of the exciting new developments that are occurring in this field.[12]

*In vitro*, IL-4 is necessary for differentiation of the naive CD-positive T-cells within the Th2 subpopulation secreting IL-4, IL-5, IL-6, IL-10 and IL-13 Although IL-4 induces IgE synthesis and enables the immediate type of hypersensitivity reaction, there is certain evidence suggesting *in vitro* and *in vivo* anti-inflammatory effects of IL-4. IL-4 is critical in switching B lymphocytes to produce IgE, for expression of VCAM-1 on endothelial cells, and for inducing the differentiation of Th2 cells and IL-5, which is essential for the differentiation of eosinophils. IL-4 is of critical importance in the differentiation of Th2 cells and is therefore an 'upstream' cytokine that is an attractive therapeutic target in the treatment of atopic diseases. Excessive IL-4 production by TH2 cells has been associated with elevated IgE production and allergy[13]

The critical role of IL-5 in eosinophilia has been confirmed by the use of an anti-IL-5 antibody in asthmatic patients, which almost depletes circulating eosinophils and prevents eosinophil recruitment into the airway after allergen. IL-5 is a cytokine that is not encountered at high levels in healthy individuals. The control of IL-5 protein production takes place at the level of transcription.[14] IL-10 is a potent anti-inflammatory cytokine that inhibits the synthesis of many inflammatory proteins, including cytokines (TNF-α, granulocyte macrophage colony stimulating factor, IL-5, chemokines) and inflammatory enzymes (inducible nitric oxide synthase) that are over-expressed in asthma.[15] In addition, IL-10 inhibits antigen presentation and sensitisation. IL-13 signals through the IL-4 receptor α-chain, but may also activate different intracellular pathways.[16]

Literature shows that Th2 lymphocytes are presently considered the main orchestrator of allergic airway inflammation underlying asthma. Functional analysis of the role of cytokines, largely based on the *in vivo* animal models, confirms this hypothesis. During T cell differentiation from naive T cells into Th1 and Th2 cells, the expression of IL-10 in Th1 cells slowly disappear, whereas Th2 cells produce more IL-10.[17]

In contrast, Th2 cells secrete IL-4, IL-5, IL-9, IL-10, and IL-13, which are involved in isotype switching of B cells as well as proliferation and differentiation into antibody-secreting plasma cells. In particular, IL-4 and IL-13 are involved in the isotype switch from IgM to IgE, the antibody responsible for classic allergy and implicated in the pathophysiology of allergic asthma. Interleukin-4 and IL-10 are also regulatory cytokines, antagonizing the activities of Th1 cytokines. Thus, the nature, intensity and duration of a specific immune response depend on the delicate balance between Th1 and Th2 numbers or activities (or both).[18,19]
